# P02.47. The relationship between CAM use and somatic symptom severity

**DOI:** 10.1186/1472-6882-12-S1-P103

**Published:** 2012-06-12

**Authors:** S Chamberlin, J Wild, D Ackerman

**Affiliations:** 1 National College of Natural Medicine, Helfgott Research Institute, Portland, USA; 2 Oregon College of Oriental Medicine, Portland, USA

## Purpose

This analysis looked at the relationship between patient self-reported use of complementary and alternative medicine (CAM) and change in symptom severity for a variety of somatic conditions.

## Methods

Data were collected prospectively through the PROCAIM Network, developed at UCLA. The Patient Health Questionnaire 15 (PHQ15) was used to measure 3 month change in symptom severity. All patients reported having chronic medical conditions characterized by chronic pain. Multivariate linear regression was used to evaluate: (1) The relationship between somatic symptom severity and the number of complementary and alternative medicine (CAM) modalities at baseline and during the three month period. (2) The relationship between somatic symptom severity and the NCCAM CAM categories.

## Results

438 patients completed the PHQ15 at baseline and three months. Lower symptom severity (PHQ15) was associated with a greater number of CAM modalities used at baseline. During the three month follow-up, an increase in symptom severity was associated with increasing numbers of CAM modalities. Lower symptom severity at baseline was associated with the use of natural products together with whole system medical approaches; natural products combined with mind body and other whole system approaches; natural products together with mind body, manipulative therapy, and whole system therapies. An increase in symptom severity during the next 3 months was associated with the same CAM usage as well as the use of natural products together with manipulative therapy.

## Conclusion

Baseline use of multiple CAM therapies, especially natural products, was associated with symptom improvement. During the next 3 month period, an increase in the use of CAM combinations was associated with increasing symptom severity. This could indicate that patients who are not improving are seeking out more CAM remedies to deal with a worsening symptom picture. This can be further evaluated with PROCAIM data for the entire 12-month follow-up period.

